# The Selectivity of Butyrylcholinesterase Inhibitors Revisited

**DOI:** 10.3390/molecules30214201

**Published:** 2025-10-27

**Authors:** Michael D. Gambardella, Yigui Wang, Jiongdong Pang

**Affiliations:** 1 Department of Chemistry, University of Pittsburgh, Pittsburgh, PA 15260, USA; 2 Department of Chemistry and Biochemistry, Southern Connecticut State University, New Haven, CT 06515, USA; 3 Department of Chemistry and Chemical & Biochemical Engineering, University of New Haven, West Haven, CT 06516, USA

**Keywords:** acetylcholinesterase, butyrylcholinesterase, Alzheimer’s disease, BChE selectivity, virtual screening, synthesis, inhibitor

## Abstract

Acetylcholinesterase (AChE) inhibitors are the primary target for single-molecule anti-Alzheimer’s disease (AD) therapeutics. Though AChE has historically been the focus of investigation for small-molecule inhibitors, interest in another cholinergic enzyme, butyrylcholinesterase (BChE), has grown in recent years. Attention stems from BChE’s role in β-amyloid (Aβ) protein aggregation and an increase in BChE concentration during the late stages of AD, where a decrease in AChE concentration is also observed. Currently, five FDA-approved drugs are on the market for inhibiting AChE, though no BChE-selective drugs have been approved so far. In this review, we focus on newly identified BChE selective inhibitors and present the ideas behind these discoveries.

## 1. Introduction

In vivo, there primarily exists two cholinesterase enzymes (ChE), termed acetylcholinesterase (AChE) and butyrylcholinesterase (BChE). Both AChE and BChE catalyze the hydrolysis of neurotransmitter acetylcholine (ACh) into choline and acetic acid, allowing the cholinergic neuron to return to its resting state following activation, albeit with differing levels of efficiency [1]. The pathogenesis of AD is believed to involve β-amyloid (Aβ) accumulation and abnormalities with tau protein hyperphosphorylation, among others [2], so the degradation of cholinergic neurons will not only impair memory and learning but also worsen the AD-related pathogenesis [3]. Therefore, targeting the cholinergic system becomes an important strategy for AD drug discovery. In healthy individuals, AChE is found in many types of conducting tissue, such as nerve and muscle tissues, central and peripheral tissues, and motor and sensory fibers [1]. On the other hand, BChE has ~160 times greater concentration than AChE, is synthesized in the liver, and is allowed to circulate in the blood, where it is present in the highest concentration in vivo [4]. As such, BChE’s primary function is to hydrolyze potentially harmful choline esters and is often used as a biomarker to track liver health [5,6]. It was found that BChE activity is significantly elevated in AD patients during advanced stages of the disease [7]. During these latter stages, AChE is known to decrease its overall concentration, limiting the applicability of AChE selective inhibitors in this period. Research has also revealed that selective BChE inhibition elevates brain acetylcholine, augments learning, and ameliorates the formation of Aβ aggregates in rodent models [8]. The inhibition of BChE is a strategy for the treatment of AD, and the discovery of selective BChE inhibitors has attracted growing interest in recent years.

**Figure 1:**
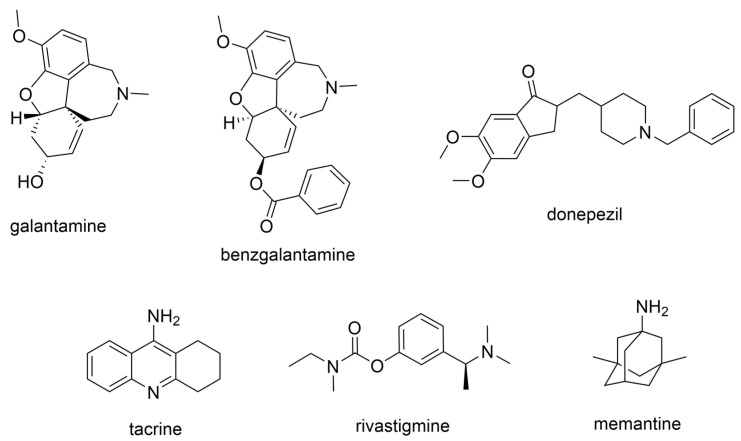
FDA-approved indirect-acting drugs for the treatment of AD, past and present.

The current U.S. Food and Drug Administration (FDA)-approved Alzheimer’s Disease (AD) single molecule inhibitors are galantamine, benzgalantamine, donepezil, rivastigmine, and memantine (Figure 1) [9,10,11,12]. The first three are indirect-acting cholinergic drugs whose mechanism of action is the inhibition of AChE. Galantamine and donepezil are AChE selective, while rivastigmine is considered a dual inhibitor. Tacrine was previously approved by the FDA for AD treatment, but it was then discontinued due to its severe side effects, including hepatotoxicity [1]. There are no FDA-approved BChE-selective drugs to date. Memantine is a therapeutic primarily used to treat moderate to severe AD due to its inhibition of N-methyl D-aspartate (NMDA) receptors in the central nervous system (CNS) [11]. More recently, the prodrug benzgalantamine joined the list of FDA-approved anti-AD therapeutics [12]. Benzgalantamine is a prodrug of galantamine and is designed to reduce gastrointestinal side effects relative to traditional galantamine. The AChE inhibitors (AChEIs) prevent the ChE from breaking down ACh by blocking the catalytic site. Reversible cholinergic inhibitors--through competitive, non-competitive, or uncompetitive mechanisms--have therapeutic applications, while irreversible cholinergic inhibitors react with enzymes to form a covalent bond with the serine of the catalytic triad and potentiate overstimulation of the postsynaptic neuron [1].

The x-ray crystallography-derived structures for the enzymes AChE and BChE provide invaluable information to assist drug discovery. AChE and BChE’s monomers are strikingly similar, having a narrow active site gorge with a widened base about 20 Å deep from the surface to the center of the enzyme. Early experimental work classified the binding gorge into esteratic and anionic subsites, corresponding to the catalytic triad and choline-binding pocket, respectively [13]. In addition to the triad at the bottom of the gorge, the esteratic site includes the acyl pocket and the oxyanion hole (Gly118, Gly119, and Val201), responsible for stabilizing the carbonyl oxygen. The anionic subsite includes the choline binding site (CBS), near the triad, aromatic residues surrounding the mouth of the gorge entrance, which involves Asp72, and near the gorge neck wall, comprised of aromatic residues Tyr121 and Phe330. The aromatic residues surrounding the gorge entrance and on the surface of the gorge are usually referred to collectively as the peripheral anionic site (PAS). Fourteen aromatic residues shaping up AChE’s active gorge hallmark the hydrophobic property of the active gorge [13,14].

In 1993, Radić et al. demonstrated that three distinct domains in AChE and BChE conferred selectivity for AChE and BChE [15]. These domains are defined by the author as clusters of aromatic residues that contribute to ChE selectivity in distinct ways. The first of these domains is known as the acyl pocket. In AChE, there exists a pair of phenylalanine residues in this domain, while in BChE, these amino acids are replaced by valine and leucine. The difference in the steric hindrance of the ChEs’ respective hydrophobic active sites allows BChE to tolerate a bulkier ligand and is the greatest difference between the two ChEs. The second domain resides near the lip of the active site, consisting of two tyrosine residues and a tryptophan, which the authors note to be critical for AChE selective bis-quaternary inhibitors [15]. The third domain is the CBS, which includes a negatively charged glutamic acid residue used to stabilize the quaternary ammonium cation of ACh. In addition, the critical tyrosine in AChE was found to block the binding site for substituted tricyclic inhibitors, such as tacrine, ethopropazine, and 9-aminoacidine, hence improving these compounds’ selectivity towards BChE.

Present-day strategies to elicit anticholinergic therapeutics with selectivity toward either AChE, BChE, or both are summarized in two directions: traditional methods and virtual screening. By traditional methods, we refer to synthesis-directed approaches of discovering new inhibitors based on known drugs, active compounds, and/or their derivatives, while virtual screening refers to the evaluation of a large database of candidate compounds through in vitro or computational methods [1,16,17]. Machine learning (ML) models have recently been incorporated into virtual screening workflows and have garnered modest success in yielding selective AChE and BChE inhibitors despite a relative lack of peer-reviewed articles attempting such studies. From the experimental perspective, quantitative high-throughput screening (qHTS), while relatively younger than classical in vitro screening methodologies, allows researchers to directly test the inhibition kinetics of an entire compound library against their target biological macromolecule at varying concentrations in efficient time scales.

## 2. Approved Therapeutics and Cholinesterase Selectivity

### 2.1. FDA-Approved Anti-AD Drugs

In the years spanning 1996–2001, traditional methods were used to discover the FDA-approved anti-AD therapeutics in Figure 1, and these drugs are, on account of their approved status, occasionally the basis for the discovery of new ChE inhibitors (ChEIs). Of these compounds, galantamine is the only secondary metabolite phytochemical approved for use against AD and dementia [18]. Galantamine, donepezil, and rivastigmine all possess a tertiary amine that is protonated at physiological pH, and the formed quaternary ammonium moiety mimics the behavior of the choline component in ACh. Tacrine, a formerly approved anti-AD drug, has fused heterocyclic aromatic/non-aromatic rings and a primary amine group, implying tacrine interacts with ChE’s hydrophobic gorge and serves as a hydrogen bond donor and acceptor. The carbamate moiety of rivastigmine reacts with the serine residue of the catalytic triad, not dissimilar to the acetyl group of ACh, though the adduct spontaneously dissociates within 30–40 min, i.e., rivastigmine temporarily forms a covalent bond with the serine residue of the catalytic triad, effectively functioning as a pseudo-irreversible inhibitor. Kinetic assay studies against human BChE (huBChE) and huAChE, as well as aryl acylamidase activities, determined that donepezil inhibited BChE through a mixed non-competitive mechanism, while galantamine inhibited through a competitive mechanism [19]. The second-order rate constant for rivastigmine is >100 times higher for huBChE than for huAChE, indicating less steric hindrance in huBChE than in huAChE for enzyme-ligand covalent bond formation. This conclusion was reached because only 6 aromatic amino acid residues lined up in the active gorge in huBChE instead of 14 in huAChE [19]. Despite this, of all the past and present FDA-approved small molecule inhibitors are selective for AChE over BChE, apart from the hepatotoxic tacrine (Table 1).

Nutho et al. studied solution behaviors of huAChE-donepezil and huAChE-galantamine complexes using molecular dynamic simulations [51]. Researchers concluded with three reasons to explain why donepezil (DPZ) is ~85 times more potent than galantamine (GNT) against huAChE: (1) DPZ showed two strong hydrogen bonds formed between the piperidine ring of DPZ and His447, as well as between the carbonyl oxygen of DPZ and Phe295 in the backbone of the ChE. In contrast, GNT showed one medium hydrogen bond occupation formed between hydroxyl group in cyclohexene moiety of galantamine and oxygen atom in carbonyl group of His447; (2) The calculations of per-residue free energy decomposition, based on the Molecular Mechanics General Born Surface Area (MM-GBSA) method, for binding of huAChE-ligand complexes indicated galantamine solely binds to residues at the catalytic anionic site (CAS), which generally refers to aromatic residues such as Trp86 and Tyr337 which surround the catalytic triad, while DPZ forms bonds to residues at the PAS. In addition, the stabilization from individual stabilizing residues was separately considered, along with the contributions from their backbone and sidechain atoms, together with the Coulombic and Van der Waals (vdW) energies. Stabilization energies were mostly contributed from side chain atoms of stabilizing residues, with the exception of Gly121, Val294, and Phe295 of huAChE-DPZ and the Gly121 and His447 residues of huAChE-GNT. Furthermore, the two aromatic residues Trp86 and Tyr337 stabilize DPZ and GNT through hydrophobic interactions, though DPZ receives extra hydrophobic vdW stabilizations from residues in the PAS; (3) Using MM-GBSA and Molecular Mechanics Poisson-Boltzmann Surface Area (MM-PBSA) methods, the total free energy of binding of huAChE-inhibitor complexes was extracted from a total of 200 molecular dynamics simulation snapshots of the last 5 ns of the production phase for per-residue decomposition calculations. Under in vacuo simulation conditions, electrostatic interactions were reported to be greater than the vdW energy by ~3-fold. However, the combined electrostatic energy in the gas phase and the solvation electrostatic free energy contribution resulted in unfavorable positive values, while the corresponding term from nonpolar contributions due to solvation enhances stabilization of the complex. The authors attributed these differences to the fact that the binding pocket is largely a hydrophobic cavity, suggesting that both vdW interaction and nonpolar solvation are key driving forces for the formation of huAChE-inhibitor complexes [51]. An interesting side point from the paper is that Glu202 significantly destabilizes DPZ through side chain dominance of Coulombic and polar characteristics, which is also true for GNT, but to a much lesser extent.

### 2.2. The Three Domains of ChE Selectivity

The first domain of ChE selectivity, known as the acyl pocket, drives selectivity through differences in aromatic residues between the ChEs. This cluster of aromatic residues lies deep within the active site gorge, where steric hindrance due to a pair of phenylalanine residues present in AChE but missing in BChE is the major distinction determining cholinergic selectivity. By restricting the active site this way, these phenylalanine residues serve to improve the efficiency of acetylthiocholine’s hydrolysis. Researchers have found that replacing these residues in AChE reduces the catalytic efficiency of acetylthiocholine while improving the catalytic efficiency of butyrylthiocholine. Residue Phe295 was found to be most restrictive toward the hydrolysis of butyrylthiocholine, and this amino acid was shown to influence acetylthiocholine hydrolysis kinetics by both decreasing the associated turnover number and K_M_ when replaced with a tyrosine residue [15]. The replacement of bulky aromatic phenylalanine residues with valine and leucine in BChE also allows certain ligands to nest a portion of their structure into the acyl pocket of BChE, practically unencumbered. The AChE active site gorge is approximately 200 Å^3^ smaller than the gorge of BChE, and a major reason for this drastic difference in binding site volume is the variance of acyl pocket residues between ChEs.

The second domain identified by Radić and coworkers resides near the lip of the active site gorge [15]. In particular, three aromatic residues, Tyr72, Tyr124, and Trp286, make up this domain. While there is no variance in these amino acids between AChE and BChE, the overall difference in size of the active site between the two enzymes makes this domain important for selectivity between the ChEs. Due to this, discussions on the second domain overlap with our discussion of the narrow gorge entrance and dual PAS and CAS sites in ChEs. Prior to Radić et al.’s paper, multiple groups had undertaken the task of introducing mutations to these aromatic residues and observed lessened binding affinity with known AChE selective inhibitors, such as BW284C51, decamethonium, and propidium [52,53,54,55]. Additionally, such mutations were found to affect the Michaelis constant for acetylthiocholine, a compound often used as a substitute for ACh when performing in vitro kinetics studies. With respect to BW284C51, mutations to these aromatic residues result in a dissociation constant nearly equal to that of BChE. Docking of BW284C51 within the AChE active site further implies energy-minimizing interactions between this compound and the trio of residues in the second domain. Another bisquaternary compound, decamethonium, experiences a similar effect, though to a far lesser extent. Researchers found that by mutating out this domain, the trimethylamino group of decamethonium becomes free to migrate to a different region in the active site. This migration is limited in the AChE complex on account of the bulky phenylalanine residues of the acyl pocket, which are missing in BChE.

Rounding out Radić et al.’s three domains of ChE selectivity is the CBS [15]. The critical residues of this domain include Trp86 and an aromatic residue at position 337, which serve to assist in the stabilization of the choline moiety of ACh. In mammalian AChE, the residue at position 337 is a tyrosine, while in *Tc*AChE, this residue is a phenylalanine. While the Trp86 residue is retained in both ChEs, in BChE, the aromatic residue at position 337 is replaced by an alanine, and this variance represents the major difference between ChEs regarding the third domain. As such, the steric clashes arising from interactions with this aromatic residue were determined to be the primary reason for ethopropazine ChE selectivity. The compounds tacrine, 9-aminoacridine, and N-methylacridinium were also evaluated against wild-type AChE, mutated huAChE (Y337A), and wild-type BChE. For all three of these compounds, results showed that the differences in inhibition were greater between the mutated AChE and wild-type AChE than between wild-type AChE and wild-type BChE. Similarly, removal of the aromatic residue at position 337 in AChE was found to yield dissociation constants closer in magnitude to that of BChE for phenothiazines, such as ethopropazine, confirming the importance of this residue for ChE selectivity. A conversation about the CBS would be incomplete without mentioning the Glu202 residue common to both ChEs. This glutamate residue is a member of the catalytic triad, and its negative charge allows for a favorable Coulombic interaction with the positively charged choline group of ACh. As this residue is found in both ChEs, attention is primarily focused on glutamate’s flanking residues when developing selective ChEIs.

### 2.3. Additional ChE Selectivity Considerations

**Figure 2:**
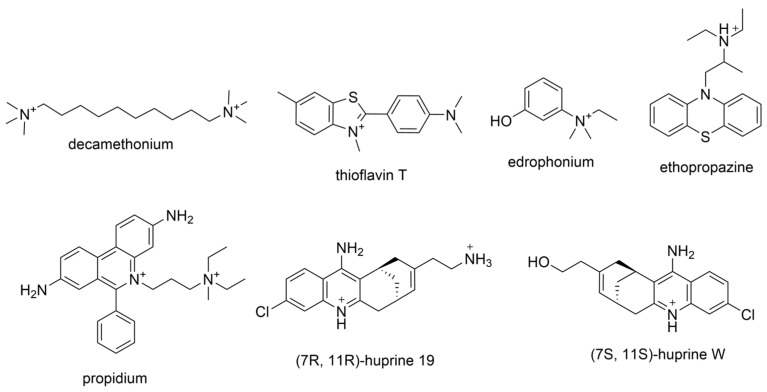
Compounds discussed for the three domains conferring ChE selectivity.

Research by Rosenberry et al. has also provided insight into cholinergic selectivity [56]. This group successfully determined X-ray crystal structures of huBChE-ligand complexes for previously determined ChEIs decamethonium, thioflavin T, ethopropazine, propidium, and (7R,11R)-huprine-19 (Figure 2), and compared ligand binding poses with those in AChE-ligand complexes. Decamethonium spans over both the gorge active site and PAS in *Torpedo californica* AChE (*Tc*AChE), where the interatomic distance between two quaternary nitrogen is reported to be 11.7 Å. In huBChE, this compound undergoes a 90°-twist, where the interatomic distance between two quaternary nitrogen is 8.7 Å [56]. Thioflavin T is not long enough to span both the PAS and CBS, and so the compound adopts a linear conformation. The dimethylamine group remained at 8.5 Å from the bottom of the gorge. Two molecules of thioflavin T filled the gorge of huBChE, with an angle of 45° between the longitudinal axes of two molecules. The dimethylamine group of one molecule interacted directly with Trp82 of the CBS, and the dimethyl group of the other occupied the acyl pocket. Propidium only binds at the gorge entrance of murine AChE (mAChE), but it binds differently to huBChE due to the absence of a residue equivalent to Trp286 in mAChE or Trp279 in *Tc*AChE. The alkyldiethylmethyl ammonium group is in proximity to the catalytic triad and forms a cation-π interaction with Trp82, while the phenanthridinium ring is nested in the acyl pocket. The authors also isolated crystals for the huAChE-(7S,11S) huprine W-complex, in which the compound matches the active site gorge through interactions with the catalytic triad and Trp86 [56]. While Rosenberry et al. hadn’t isolated a similar crystal structure in the huBChE complex, they instead successfully isolated the crystal of huBChE-(7R,11R)-huprine-19 complex. For context, huprine derivatives are strongly AChE selective. Ethopropazine is a substituted phenothiazine with a marked BChE selectivity over AChE by a ~9000-fold difference in KI values. The phenothiazine ring is slightly bent, and one ring extends into the acyl pocket, where the oxyanion hole, represented by residues Gly116 and Gly117, still holds enough space for water molecules to inhibit. The location of the phenothiazine ring depends on Tyr332 and Phe329, whose side chains project into the active site gorge. The authors note that the ability of the acyl pocket in BChE to accommodate aromatic rings without any conformation change stands remarkably in contrast to that of AChE. The major interaction site of the tested polycyclic aromatic compounds in AChE is the cluster of aromatic residues at the PAS. The authors suggest that targeting the acyl-binding pocket for designing BChE inhibitors is a worthwhile strategy to follow.

**Figure 3:**
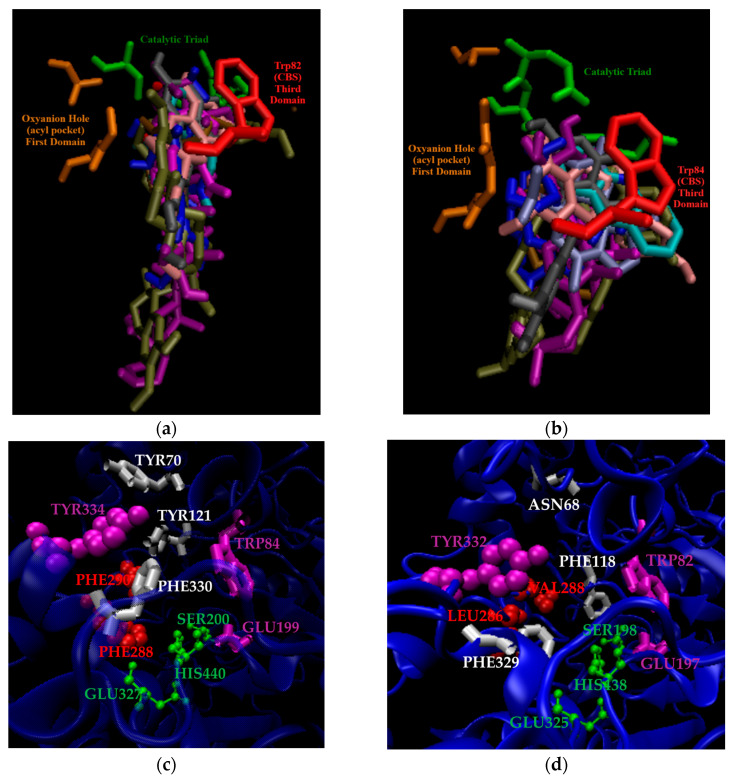
The most favorable poses of eight secondary metabolites in the AChE (PDB: 1W6R) and BChE (PDB: 4BDS). (**a**) 1W6R-ligand complexes; (**b**) 4BDS-ligand complexes; Color codes for proteins: Green—catalytic triad residues; orange—oxyanion hole residues; red—Trp82 or Trp84 residue of the CBS; and white—aromatic side chains of residues. Color codes for ligands: cyan—co-crystalized ligands (galantamine in AChE and tacrine in BChE); pink—afzelechin; blue—aspalathin; purple—D-maslinic acid; ochre—isoliensinine; gray—luteolin; ice blue—matricin; tan—sedanolide; magenta—thebaine. (**c**) three domains in AChE (1W6R); (**d**) three domains in BChE (4BDS). Color codes: green—catalytic triad; red—representative residues for the first domain; white—representative residues for the second domain; magenta—representative residues for the third domain.

Recently, our group used molecular-docking-based virtual screening against the ZINC FDA-approved (1614) and metabolite-in-vivo-clean (37,091) datasets for both ChEs [57]. The most targeted residues by atoms of ligands in our datasets were tabulated, and frequency analysis of docking-predicted selective ligands was performed. The datasets’ ligands’ hydrogen atoms were found to primarily target the hydroxyl or carboxylate functional groups in the side chains of Ser198, Glu197, and Tyr128 of BChE. Similarly, the hydrogens of the datasets’ ligands were clustered near AChE residues Glu199, Tyr121, and Ser122. The oxygens of the datasets’ ligands favored the hydrogen atoms attached to BChE residues Gly116, Trp82, and Thr120, and AChE residues Ser122, Ser200, and Tyr121. The nitrogen atoms of the datasets’ ligands targeted the hydroxyl groups in the side chains of AChE residues Tyr121, Ser122, and Glu199, while the ligands’ nitrogen targeted BChE residues Gly116 and Thr120 most frequently. The high frequency of the datasets’ ligands’ oxygen and nitrogen atoms targeting the Tyr121 residue of AChE further quantifies the importance of the PAS for ChE selectivity, from a structural perspective. There was a slight favorability of our ligands in these test sets to be selective toward BChE, and selective BChEIs were found to have a greater difference in binding affinity than docking predicted selective AChEIs due to clashes with the bulky aromatic residues of the acyl pocket in AChE. In lieu of studies in vitro, we selected a number of FDA-approved and secondary metabolites for 100 ns molecular dynamics production runs in triplicate against both ChEs based on the docking-predicted difference in binding affinity, docking-predicted upper and lower bound RMSDs, known therapeutic windows, the Lipinski Rule of Five, and/or known ChE inhibition metrics [58]. The most energetically favorable binding poses of eight of these compounds in *Tc*AChE (PDB: 1W6R) and in equine BChE (eqBChE) (PDB: 4BDS) are shown in Figure 3. One of these compounds in silico results, D-maslinic acid, shows the highest potential for BChE selectivity due to docking analysis, Gibbs free energy of binding calculations, RMSF of active site residues, and increased unfolding of AChE relative to the uninhibited enzyme when in complex with the compound, likely due to steric clashes with the pair of phenylalanine attributed to the primary domain of AChE and residues of the PAS.

## 3. Selective BChE Inhibitors from Traditional Methods

### 3.1. Derivatives of Galantamine (GNT), Donepezil, Tacrine, and Rivastigmine

**Figure 4:**
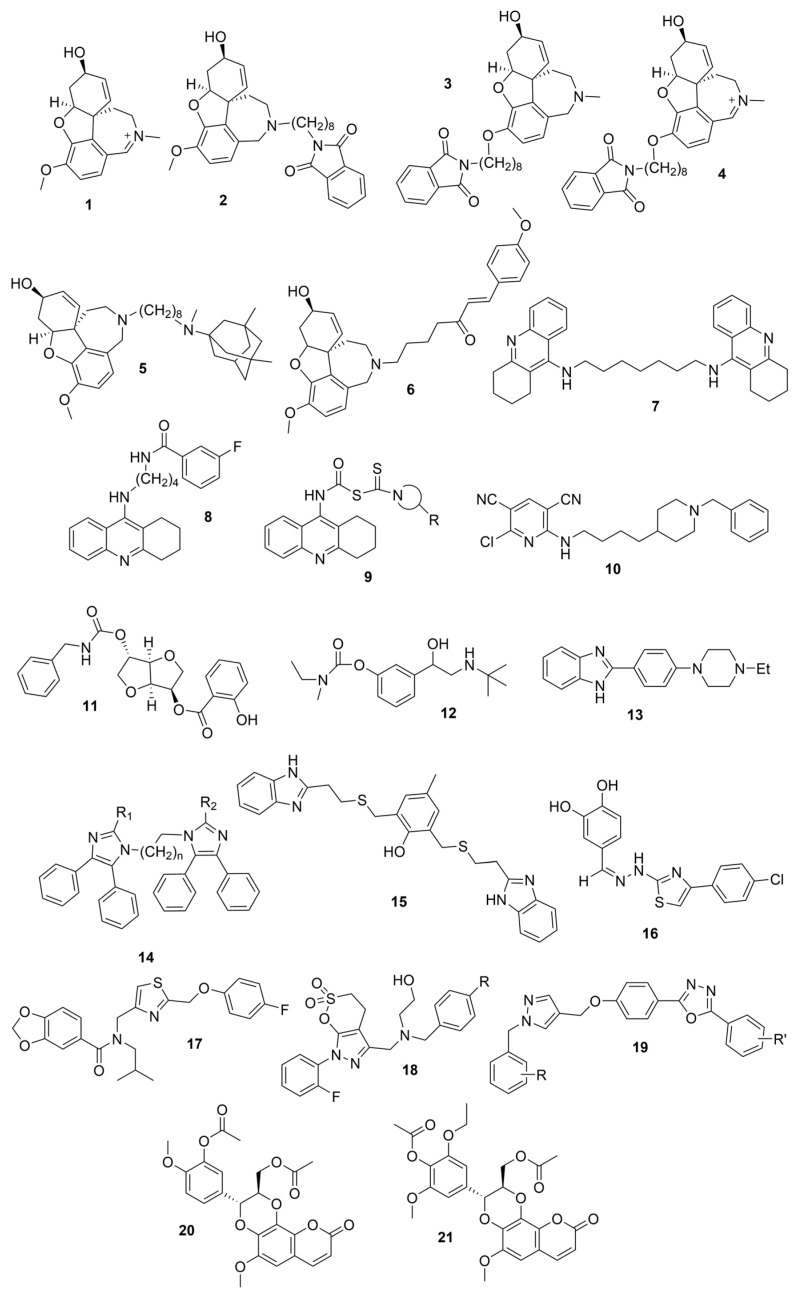
Derivatives of galantamine and BChE inhibitors identified via traditional drug discovery methods.

Galantamine is known to be selective for AChE over BChE and has garnered the most attention of all currently approved anti-AD therapeutics for the development of novel and selective BChEIs. A series of GNT analogues (**1**, **2**, **3**, and **4**) identified by Bartolucci et al. and their biological activity (Figure 4 and Table 1) exemplify the structural impact on inhibitory potency. In vitro *Electrophorus electric* AChE (*Ee*AChE) kinetics testing revealed that the IC_50_ values for these ligands are 0.36 µM, 0.14 µM, 0.28 µM, 2.50 µM, and 0.07 µM for GNT, **1**, **2**, **3**, and **4**, respectively [20]. Firstly, the authors note that the permanent quaternary moiety in **1** and **4** increases the inhibitor potency more than the tertiary amine group in GNT and 3, respectively. Secondly, a large substituent attached to the tertiary amine group through an alkyl linker significantly enhances GNT’s inhibitory potency as seen in compound **4**. The bulky substituent at the methoxy site of GNT dramatically decreases GNT’s inhibitory potency, as shown in compound **3**. Bartolucci et al. had also obtained the *Tc*AChE-GNT crystal structure at 2.5 Å resolution through x-ray crystallography [20]. The authors noticed that GNT did not directly interact with Trp84 but still lies within the principal quaternary ammonium binding site, emphasizing that inhibitors might orientate differently due to variance in 3D-structure compared to ACh.

Greenblatt et al. additionally obtained 3D X-ray crystal structures for *Tc*AChE-inhibitor complexes for **1** (PDB: 1W6R) and **4** (PDB: 1W76) and the quaternary analogue of **2** (PDB: 1W4L) [59]. The crystal structure and docking results demonstrated that the quaternary ammonium group on **2** analogues positioned toward the choline binding site, while the phthalimido moiety attached to N via an alkyl linker interacts with the PAS site, thereby creating the bis-interacting effects. The hybrid of GNT and memantine produces multitargeted chemical **5**, which is much more potent than GNT alone [21]. The bulky substitute for the methoxy group reduces the GNT activity toward AChE. The x-ray-derived crystal structure revealed that the GNT moiety is still positioned similarly in *Tc*AChE-**4** crystal as in *Tc*AChE-**1**. However, the added phthalimide moiety is noted to pierce the Trp-276-Ser291 loop of the protein, which then protrudes into solvent. We have not noticed many other cases of BChE selective inhibitors designed by modifying the MeO moiety of GNT in the literature. On a different note, benzgalantamine, which is synthesized via modification at GNT’s hydroxyl group, was recently approved by the FDA for anti-AD and found to reduce gastrointestinal side effects relative to GNT [12].

In line with the notion of dual-site inhibition, a GNT hybrid derivative with another natural product, curcumin (CCN), at the tertiary amine site (**6**), was newly synthesized and reported to outperform its parents. Specifically, **6** significantly inhibits BChE in murine brain homogenates measured ex vivo, where scopolamine-induced neurotoxicity was assessed [22]. Salamanova et al. explored conformation flexibility of GNT, CCN, and **6** in a homology model of mBChE using 1 µs molecular dynamics simulations in triplicate [60]. The authors clustered molecular conformations from an MD trajectory by grouping similar conformations based on differences in spatial coordinates or energies. All three compounds were found to form stable complexes with mBChE, and the backbone atoms of the enzymes remained stable throughout the simulations. Eight hydrogen bonds persisted for GNT over 100 ns during 1 µs simulations, three unique hydrogen bonds persisted for CCN, and six unique hydrogen bonds persisted for compound **6** during the same time. In the case of compound **6**, His435 of the catalytic triad was found to form two long-lived hydrogen bonds with O_6_′. The GNT moiety of **6** is positioned differently in the binding site compared to GNT alone, so that N12 forms a hydrogen bond with Ser284. Additional carbon-hydrogen bonds were observed between **6** and the side chains of BChE [22]. Unfortunately, no parallel comparison is available for AChE, and thus, the BChE selectivity over AChE for **6** is unknown.

Tacrine has been co-crystalized with the enzymes huAChE (PDB: 1ACJ) [61] and huBChE (PDB: 4BDS) [62]. Compound **7** with two linked tacrine molecules has an IC_50_ of 0.4 nM for mAChE [23], demonstrating **7**’s multitargeted inhibition at both the PAS site and CAS site, which enhances the inhibitor’s potency for AChE. Bis-tacrine has many biological activities, such as inhibiting both ChEs, acting as an antagonist toward NDMA, inhibiting nitric oxide synthase, and mitigating Aβ aggregation [63]. Compound **8** was found to be more potent towards BChE than AChE [24]. A series of 2-(9-acridinylamino)-2-oxoethyl-piperazine/piperidine/morpholine-carbodithioate derivatives (**9**) were synthesized, and the compound with a N moiety, being a 4-[4-(trifluoromethylbenzyl)-piperazinyl group, demonstrates BChE selectivity [25]. The kinetic assay studies showed a mixed inhibition mechanism, a potential indicator that **9** may bind to both the CAS and PAS. Molecular docking revealed a π-π interaction between His438 of the catalytic triad and the benzene ring, similar to tacrine in complex with BChE, and a hydrogen bond between the -CF_3_ group on the benzene ring and Gly115 of the oxyanion hole. The representative anionic residue Trp82 lies in proximity to the piperazinyl ring, while the planar tricyclic acridinyl moiety is bent out toward the PAS. The authors attributed BChE selectivity to interactions with the catalytic triad, though there have not been any parallel studies against AChE. Compound **9** was found to be nontoxic against the murine fibroblast cell line (NIH3TS) with an IC_50_ > 1000 µM, and can permeate the blood-brain barrier (BBB).

Rivastigmine is a dual inhibitor with a carbamate moiety. It inhibits ChEs by reacting with the serine residue at the catalytic triad, then the carbamyl moiety splits from the enzyme via spontaneous hydrolysis [1]. Compound **11** is BChE selective with an IC_50_ of 0.15 nM for huBChE, which is 666,000 times more potent than for huAChE [23]. The high potency is due to an interaction between the hydroxyl group of the 5-salicylate moiety and the polar Asp70/Tyr332 cluster in the PAS of BChE, in addition to the carbamate moiety that has pseudo-substrate interactions with the active site [26]. Mutant studies confirmed the important role of Asp70 and Tyr332 in forming a hydrogen bond with the hydroxyl group of **11**. In another publication, which was inspired by the fact that the structures of rivastigmine and bambuterol are similar but their selectivity for BChE and AChE differ, a hybrid rivastigmine-bambuterol compound (**12**) and fourteen of its analogues were synthesized [27]. Compound **12** was found to be more selective for eqBChE, with an IC_50_ > 100 µM for *Ee*AChE. When the quaternary carbon terminal group of **12** was replaced by an azo-cyclohexane group (MRT-3), the inhibition potency was increased by ~5-fold. The difference in the CBSs between BChE and AChE contributes to the high specificity of **12** and analogues for BChE, i.e., the ethanolamine sidechain in these compounds and bambuterol play a key role in their differing BChE selectivity. In silico chemical analysis confirmed that molecular properties, such as MW, ability to cross the BBB, and number of hydrogen bond acceptors and donors, for **12** and its analogues, are within the appropriate range based on Lipinski’s Rule of Five. The authors also measured the inhibition constant and decarbamylation rate constants for MRT-3. No further computational analysis was pursued to our knowledge.

Donepezil, on the other hand, is strongly AChE-selective (Table 1). New dual AChE inhibitors were synthesized, and compound **10** has a reported IC_50_ of ~13 nM for human AChE. However, compound **10** is 625-fold more selective for huAChE than for huBChE [28], suggesting that a proper dual-site inhibitor is more likely to be an AChE-selective inhibitor. We did not find many BChE selective inhibitors based on DPZ derivatives.

### 3.2. Derivatives from Imidazole, Thiazole, and Other Pharmacophores

Some benzimidazole derivatives have the potential for anti-AD activity due to high affinity to Aβ aggregates and high uptake into the brain [64]. Recently, a series of benzimidazole derivatives was synthesized and tested against ChEs. It was found that compound **13** has a BChE selectivity index of 6.7 [29]. Kinetic studies showed that **13** was a partial non-competitive inhibitor for BChE, and molecular docking calculations revealed that the tertiary amine ring of **13** reached the CAS, represented by its proximity to His438 and Trp82 residues, while the benzimidazole moiety interacted with the PAS site, represented by Asp70 and Tyr332. The cause for BChE selectivity was not explained, though the interactions between the central phenyl moiety of **13** and AChE may provide clues. Due to its ability to interact with both the CAS and PAS, **13** is a multitargeted inhibitor with a rigid linker. Compound **13** was experimentally found to exert a potential neuroprotective effect against hydrogen peroxide and Aβ-induced cytotoxicity in SH-SY5Y cells in vitro.

**Figure 5:**
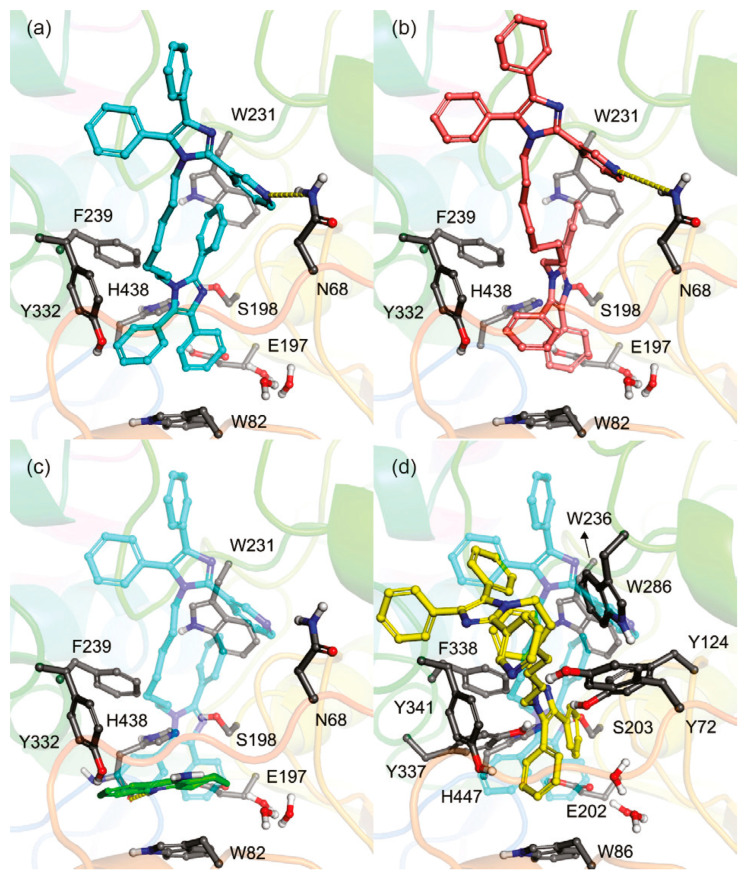
Predicted binding modes of the compounds (**a**) **14** (*n* = 7, carbon atoms colored cyan), (**b**) an analogue (*n* = 8, carbon atoms colored red-orange), and (**c**) tacrine (carbon atoms colored green) superimposed to **14** against BChE (PDB: 6I0C). (**d**) Predicted binding mode of the compound **14** against AChE (carbon atoms colored yellow, PDB: 1Q84) superimposed with its binding mode predicted against BChE (transparent sticks with carbon atoms colored cyan). Hydrogen bonds are represented as yellow dashed lines [30]. Reproduced with the permission of Prof. Marco Antonio Ceschi.

A series of dual-site inhibitor bis(*n*)-lophine (**14**, *n* = 6,7,8, where R^1^ and R^2^ are phenyl and pyridyl groups, respectively) was synthesized through one-pot four-component reactions [30]. Compound **14** analogues possess two benzimidazole moieties functioning as dual-site inhibitors. Analogue **14**, when *n* = 7, R^1^ = Ph, and R^2^ = 3-Py, was selective for eqBChE with an IC_50_ in the nanomolar range. Interestingly, all the compounds evaluated in this study were found to be inactive for *Ee*AChE. Kinetic studies indicated a non-competitive inhibition mechanism, and molecular docking in BChE (PDB: 6I0C) and AChE (PDB: 1Q84) demonstrated that **14** (*n* = 7, R^1^ = Ph, and R^2^ = 3-Py) comfortably extended over both CAS and PAS sites in BChE, while twisting awkwardly to avoid steric clashes with Trp286, Trp236, Tyr72, and Tyr124 residues in AChE (Figure 5). Two representative analogues of **14** showed no measurable cytotoxic effects in kidney Vero, hepatic HepG2, and C6 astroglial cell lines, but a large MW of ~690 Da for **14** violates Lipinski’s Rule of Five for a drug-like candidate.

Another benzimidazole **15** was successfully identified as a BChE selective inhibitor through a combination of in silico/in vitro methodologies. Compound **15** was found to be a potent inhibitor of BChE and is almost inactive for AChE [31]. Molecular docking suggests that compound **15** achieves its nanomolar potency via several π-stacking interactions with both Phe329 and Tyr332 residues in BChE (PDB: 4BDS) from its dual benzimidazole moieties. Selectivity for BChE was attributed to the inhibitor clashes with several aromatic residues, primarily Tyr124, Phe297, and Phe337, in the binding site of AChE (PDB: 4EY5). Like tacrine, compound **15** does not possess a typical choline-like cationic group at physiological pH, so the major interactions are comprised of π-stacking and hydrophobic interactions. Compound **15** is quite linear compared to other compounds considered, with many rotatable bonds, and as such, it more easily adopts a bent conformation to take advantage of the hydrophobic property of the active gorge in both BChE and AChE. At the same time, **15** remains sterically hindered from settling in AChE’s acyl pocket.

Thiazole is an important class of heterocyclic compounds found in many potent biologically active molecules, such as thiabendazole, an anthelmintic drug; riluzole, an anticonvulsant drug; and talipexole, an anti-Parkinson’s drug. This class is also involved in drug development for the treatment of inflammation, and members of this class are used as anti-glycating agents and as anti-diabetic agents. Furthermore, thiazoles have been found to be a potent inhibitor of both ChEs. The design and synthesis of novel thiazole derivatives was reported, and **16** is one representative compound that demonstrates selectivity for BChE over AChE [32]. Compound **16** is able to contort its structure to form five hydrogen bonds and π-stacking interactions with five residues at the active gorge of BChE, especially a tryptophan residue that overlaps over the top of the molecule.

In another article, compound **17** and 33 analogues were synthesized. Besides thiazole, compound **17** possesses four other functional moieties [33]. Compound **17** was found to be a sub-µM dual inhibitor, slightly favoring BChE. The authors report that in the BChE-**17** complex, the thiazole ring acts as a proper linker to aromatic rings at position two to form π-alkyl and π-π interactions with several residues in the active gorge. The δ-sulfone-fused heterocycles may be efficiently synthesized through a click chemistry reaction from sulfur (VI) fluoride exchange (SuFEx). The potency and selectivity of *δ*-sulfone-fused pyrazoles as BChE inhibitors can be significantly improved through the introduction of a tertiary benzylamine, following the hypothesis that the tertiary benzylamine group can effectively mimic the tertiary amine group of donepezil. Compound **18** (R = Br) was then synthesized and measured to be >2000-fold more selective for BChE over AChE [34]. Molecular docking analysis suggests that the δ-sulfone ring lies in proximity to triad residue His438, while the pyrazole moiety, with its phenyl group, interacts with Trp82 at the CBS, and the structure of p-Br-benzylamine is bent to interact with residues at the PAS.

Both triazole and oxadiazole are synthetic heterocyclic moieties showing potential as cholinesterase inhibitors. A total of twenty 1,2,3-triazole-methoxyphenyl-1,3,4-oxadiazole derivatives were synthesized using microwave-assisted organic synthesis [35]. Compound **19**, where R = 4-CH_3_ and R’ = 4-NO_2_, is reported to be highly BChE selective. Kinetics studies revealed a competitive inhibition mechanism for this compound when in complex with eqBChE. Trp82 residues of BChE were found to form three π-π stacking interactions with the phenoxy linker and 4-methylbenzylthiozole-1,2,3-triazole moiety of **19**. Ser198 and His438 residues of the catalytic triad separately contributed a hydrogen bond and a π-π stacking interaction with the 1,2,3-oxadiazole ring of **19**. The peripheral residues formed two π-cation interactions and one π-π stacking interaction with the nitro and nitroaryl groups of **19**. Compound **19** adopted a U-shaped conformation, and only one π-π stacking interaction with Tyr124 in the AChE-**19** complex was observed. During 100 ns dynamics simulations, unbound BChE maintained an RMSD value of ~1.6 Å throughout, while the BChE-**19** complex displayed instability in the first 20 ns but stabilized at 0.6 Å afterwards. On the contrary, the AChE-**19** complex showed higher instability than unbound AChE with RMSD values at ~2.5 Å after 35 ns of simulation. The RMSF plots demonstrated that the BChE-**19** complex reduced fluctuations of residues at the PAS and of the catalytic triad, while the AChE-**19** complex was not as effective in reducing such fluctuations.

Previously, two coumarinolignans were isolated from the root of the West African shrub *Allophylus spicatus*. From these compounds, Okpala and coworkers synthesized coumarinolignans, **20** and **21,** via an acetylation reaction using acetic anhydride in pyridine (Figure 4) [36]. Both of these compounds yielded IC_50_ values sub-60 µM against BChE, though parallel studies against AChE hadn’t been performed. As such, direct knowledge of the selectivity of these compounds for BChE over AChE is unknown. Despite this, compounds **20** and **21** interact with Trp82 at the CBS and residues His438 and Ser198 of BChE’s catalytic triad. These compounds are also able to extend to the acyl pocket, as identified through follow-up in silico experimentation. Their bent conformations overlap well with decamethonium in complex with BChE (PDB: 6EP4). The RMSD of the protein backbone revealed equilibration before 10 ns and minimal variations during 100 ns simulations for both complexes and the apoprotein. The complexes had lower per-residue RMSF values than the unbound protein, while radius of gyration and solvent accessible surface areas plots did not show much volatility during the simulations. Lastly, per residue free energy of binding analysis indicated an important contribution from Trp82 in the complexes, potentially indicating these ligands favor BChE over AChE.

Flavonoids are a class of polyphenolic compounds, which are found in many plants, and graveolinine is a 2-phenyl quinolone alkaloid isolated from *Ruta graveolens* L. Following multi-targeted inhibitor design, i.e., inhibitors designed such that they interact with both CAS and PAS, BChE-selective inhibitors were also obtained as 4-dimethylamine flavonoid derivatives and graveolinine derivatives [65]. The BChE selectivity index, given by IC_50_(BChE)/IC_50_(AChE), is usually less than five for these newly found BChE selective inhibitors on account of a missing linker to clash with the PAS residues of AChE.

## 4. Selective BChE Inhibitors from Virtual Screening

### 4.1. Structure-Based Virtual Screening

**Figure 6:**
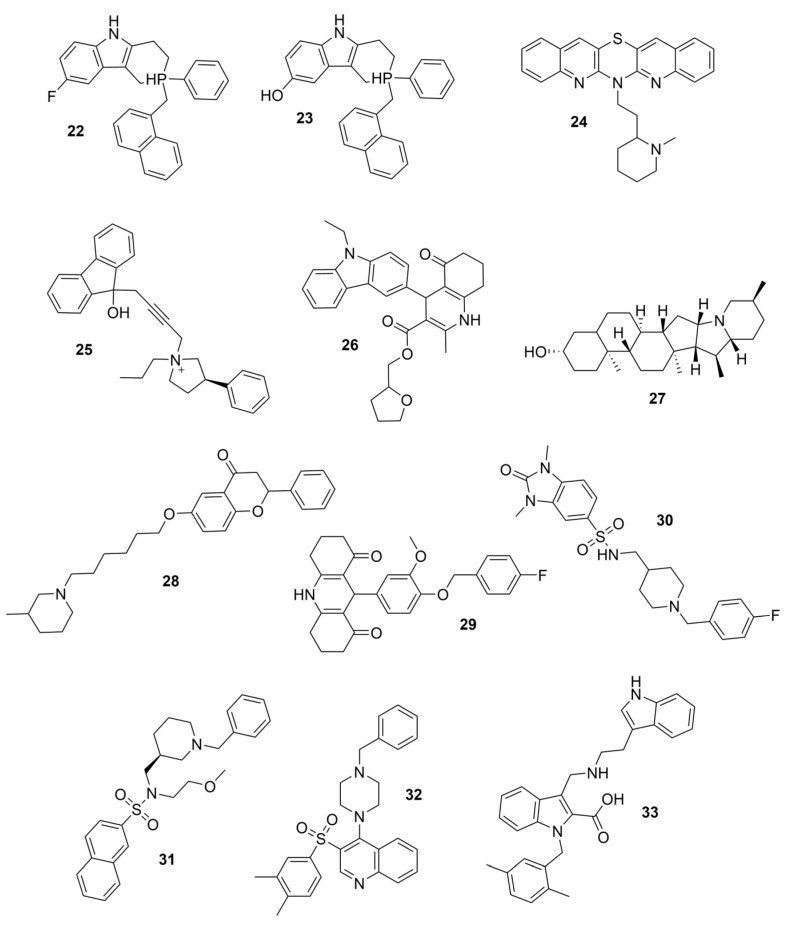
BChE inhibitors and other compounds identified from SBVS and LBVS.

Structure-based virtual screening (SBVS) makes use of the 3D structure of a target biological macromolecule to help identify potential intermolecular binding interactions that may occur within a given protein-ligand complex for a dataset of candidate inhibitors. In one such study, Lodarski et al. selected 143 heterocyclic molecules from an in-house compound library derived from the azaphenothiazine scaffold for SBVS via molecular docking [37]. In particular, these compounds were docked into the active sites of AChE (PDB: 1ACJ) and BChE (PDB: 2CKM). Fifteen compounds were then selected for in vitro biological tests, and of these, five compounds displayed moderate AChE and good BChE inhibitory activity at screening concentrations of 10 mM. The IC_50_ values for the active BChE inhibitors are in the ~11.8–122.2 nM range, where compound **24** has the lowest IC_50_ (Figure 6, Table 1). At 10 mM concentration, **24** inhibits enzyme activity 99.76 ± 0.03% for BChE and 34.77 ± 0.31% for AChE. Kinetics studies indicated a competitive inhibition mechanism of binding for this compound. In the BChE-**24** complex, the pentacyclic ring interacts with the Trp82 residue, and the protonated amine group of the N-methyl-2-piperidinethyl group forms a hydrogen bond with the hydroxyl group of the Ser198 residue of the triad. Three of the most active inhibitors possess either tetra- or pentacyclic derivatives of azaphenothiazines, which may enhance the BChE selectivity.

**Figure 7:**
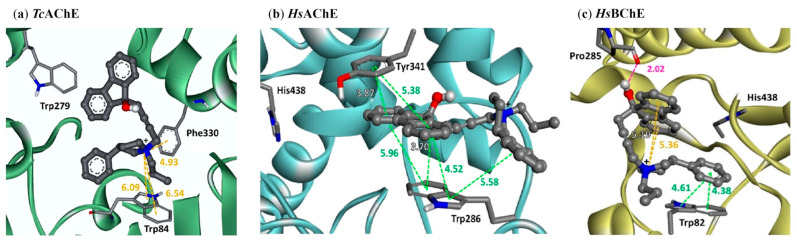
Interactions for compound **25** with AChE and BChE [38]. Reproduced with permission from Prof. João Batista Teixeira Rocha.

Since the choline group of ACh is a quaternary ammonium group at biologically relevant pH, some researchers believe compounds with such a functional group might have a better chance of inhibiting ChEs. Nogara et al. filtered the ZINC compound library to obtain 382 compounds both bearing a quaternary moiety and obeying Lipinski’s Rule of Five [38]. These compounds were then docked within the active binding site of nine AChE crystal structures. Virtual screening provided seven hits for follow-up in vitro inhibition analysis against three AChEs and one BChE. Compound **25** has a sub-µM IC_50_ for eqBChE, and IC_50_ values of 92–762 µM for the three AChE enzymes. Notably, **25** has only two cation-π interactions with the Trp84 residue in the *Tc*AChE-**25** complex, while in the huAChE-**25** complex, **25** has a larger number of π-π interactions with Tyr341 and Trp286 at the PAS. For the huBChE-**25** complex, **25** shows a strong hydrogen bond with Pro285, and the π-π and cation-π interactions occur between **25** and residues in the anionic subsite (Figure 7). Binding affinity values from docking did not correlate well with experimental IC_50_ values, with binding affinities of −11.8 kcal/mol, −9.9 kcal/mol, and −10.6 kcal/mol for the *Tc*AChE-**25** complex, huAChE-**25** complex, and huBChE-**25** complex, respectively.

As tertiary amino groups are important for cation−π interactions with active site residues of ChE and have reasonable BBB permeability, Dighe et al. performed virtual screening on 567,981 molecules from CoCoCo-SC Asinex and ChemBridge CNS Set databases, giving preference to tertiary amine molecules that also obey Lipinski’s Rule of Five [39]. The authors further required π-π interactions between molecules and Trp231, Trp82, and Phe329, intermolecular hydrogen bonding of the molecule with His438, and entry of the molecule within the acyl pocket. After follow-up docking against ChEs, 13 molecules were selected for in vitro inhibition testing against BChE and AChE. Of this pool, one compound, compound **26,** was identified as a sub-µM selective BChE inhibitor and is reported as inactive for huAChE at 10 µM [39]. Compound **26** was co-crystalized with huBChE in a 2.8 Å resolution crystal structure. The carbazole ring of **26** resides in the acyl pocket, forming π-π interactions with Trp231 and Phe329. The primary amine of the hexahydroquinoline ring forms a hydrogen bond with His438, and the hexahydroquinoline ring has π-π interactions with Trp82. Also, the R-isomer of the carbon stereocenter bearing the carbazole ring better fits the electron density than the S-isomer [39]. The tetrahydrofuran ring best overlaps with the carbazole ring in the acyl pocket through a bent conformation. The authors note the bulky carbazole ring of **26** resides in the acyl pocket of BChE, and it is considered the major difference between BChE and AChE in terms of inhibitor **26**’s BChE selectivity.

Natural products may often arise as hits when performing SBVS. For example, Zhou et al. performed virtual screening on the Development Therapeutics Program (DTP) Release **4** compound library containing ~265,000 compounds [40]. The authors utilized AutoDock Vina (v1.1.2) to perform virtual screening and docked the library against both huAChE (PDB: 4EY5) and huBChE (PDB: 4BDS). After docking, selective BChE inhibitors from natural products with solanaceous alkaloid scaffolds were identified, followed by in vitro activity assays for the top 10 compounds. Three of these tested compounds were the most potent BChE inhibitors, among which solanidine (**27**) had an IC_50_ of ~17 nM for huBChE, and **27** exhibited less than 20% inhibition of AChE at 5 μM concentration. The docking poses suggested that compound **27** was relatively rigid, forming a hydrogen bond with His438 of the catalytic triad of BChE. However, the same pose introduced clashes with Tyr124, Trp286, and Tyr337 in the more enclosed active site of AChE.

As previously discussed, multi-target-directed ligands are an interesting and promising approach to the design of selective ChEIs. Bajda et al. used docking-based virtual screening to filter a non-imidazole histamine H_3_ receptor ligand library [41]. All selected ligands were able to bind to both the CAS and PAS sites of BChE and AChE. The most promising derivatives combined the flavone moiety via a six-carbon atom linker with a heterocyclic moiety. Compound **28** has sub-µM IC_50_ values for both ChEs, though it marginally favors BChE over AChE. Though **28** is highly potent, it is not highly BChE selective. Such results further the notion that multi-target candidates will lack in BChE selectivity due to favorable interactions with the CAS and PAS of AChE, unless they possess a relatively bulky linker.

SBVS was performed on 64,124 amine-containing moieties from two compound libraries, and 9-phenylacridinedione (9-PAD) was identified as a promising scaffold for selective inhibition of BChE [42]. Joubert and Kapp then extracted 1,374 9-PAD-containing compounds from the ZINC database and then docked this compound library with huBChE (PDB: 4BDS) and huAChE (PDB: 4EY7). Seven compounds were selected for in vitro analysis of eqBChE and *Ee*AChE. The docking pose of one of these compounds (**29**) in huBChE suggests π-π stacking with Trp231 at the CBS and a hydrogen bond between the amino group and His438 of the catalytic triad. The critical Trp82 residue was also in proximity to the 9-phenylacridinedione ring, likely forming a π-stacking interaction with this residue of the CBS. Despite compound **29**’s inability to dock cleanly into AChE, the kinetics results indicate **29** has a greater affinity for AChE over BChE.

Jiang et al. have also used virtual screening to identify novel selective BChEIs via a SBVS methodology [66]. Researchers began with a database of 1225 compounds with varying scaffolds. These compounds were then screened against BChE (PDB: 4BDS), and the top 100 compounds were visually analyzed and clustered using Pipeline Plot 7.5. From this, 30 molecules were chosen for an in vitro AChE and BChE assay. The most potent of the compounds was chosen for follow-up structural optimization, where 30 new molecules were synthesized. Of these, two were found to be selective for BChE over its sister ChE. A combination of molecular docking, molecular dynamics, and in vitro kinetics experimentation reveals that these two ligands are capable of targeting both the CAS and the PAS of BChE. In addition, researchers were able to show that these compounds effectively decreased Aβ aggregate formation via a ThT-based fluorometric assay and exhibited nontoxicity against SH-SY5Y cells.

A group of researchers predominantly based in Australia utilized SBVS in tandem with physicochemical filtering to discover a novel BChEI [67]. These researchers utilized both the Maybridge and InterBioScreen libraries as their dataset, totaling 595,284 compounds. PAINS compounds and predicted aggregators were removed, and priority was given to compounds with significant CNS and HumanOralAbsorption values as predicted by QikProp. The resultant 22 molecules were then evaluated in vitro, where it was found that while many were BChE selective, only one was deemed meaningfully potent enough to continue analysis. This compound was found to have two primary interactions, which were further investigated with 50 ns molecular dynamics simulations. These interactions involved a π-π stacking interaction with Trp82 and an electrostatic interaction between the quaternary ammonium group of the benzylpiperazine and Asp70. Such interactions at the PAS and CBS were supported by observed π-π interactions with Phe329 and intermolecular hydrogen bonding with Tyr332.

Zhou et al. relied on SBVS to assist in their identification of a selective BChE inhibitor [68]. Researchers used the Enamine CNS library and ChemDiv CND-MPO library and utilized molecular docking to whittle down their pool of compounds to 84. The remaining ligands underwent MM-GBSA, and eight of the nine molecules with estimated Gibbs free energy of binding less than or equal to -90 kcal/mol were purchased for kinetics evaluations. Of these ligands, all of which were not potent AChEIs, one was found to be especially selective for BChE. The compound is believed to inhibit the CAS, where its imidazole ring forms strong π-π stacking interactions with Trp82. An intermolecular hydrogen bond was observed between Thr120 and the 1,4-diazapan-2-one ring’s 2-carbonyl group, while the phenyl group occupies the acyl binding pocket in BChE.

Chen et al. conducted VS on the ChemDiv compound collection, which contains 1,293,896 molecules [43]. Initially, a 3D model was created using the AChEI donepezil as a template, and the collection was screened by rapid overlay of chemical structures (ROCS). Then, the top 1% of the most similar structures were screened by a structure-based pharmacophore model based on the AChE-DPZ crystalized complex. Finally, 24 of the best hits were tested for AChE and BChE activity in vitro by Ellman’s method. Five new ChE inhibitors with unique scaffolds were discovered. Compound **28** exhibited the lowest nanomolar range in IC_50_ and the highest selectivity against BChE. Compared to the conformation in AChE, **30** exhibited a contracted U-shaped pose in BChE to effectively interact with the catalytic triad and peripheral residues. If **30** were to use this same conformation to bind with AChE, two collisions occur: one involving the sulphanilamide moiety, and the other occurring at the phenyl ring [43]. Interestingly, the tertiary amine of **28** interacts less significantly than it does in the case of DPZ.

### 4.2. Ligand-Based Virtual Screening (LBVS)

LBVS is a technique based upon the principle that chemical compounds with similar properties often possess similar biological activity. LBVS for ChEIs occasionally employs pharmacophore hypotheses consisting of the structural features common to FDA-approved ChEIs or other known inhibitors, including an ionizable amino group for salt-bridge, cation−π interactions in the active site, aromatic rings for π-π interactions, and hydrogen bond acceptors and donors. To date, the chemical scaffolds for approved ChE inhibitors have been relatively limited. In search of more BChE inhibitor scaffolds, Lu et al. created and validated several pharmacophore hypotheses by a ROCS spatial feature model starting from the complex of BChE and **31** (PDB: 5DYW) [44]. The model with the best performance was used as a three-dimensional entry to search several commercial databases. Compound **32** was identified as the most potent BChEI, with an IC_50_ of 1.3 μM. The in vitro kinetics of BChE indicated a competitive mechanism of inhibition, which was supported by MD simulations. Yang et al. used the same pharmacophore model to screen the combined Life Chemicals, ChemDiv, Enamine, and InterBioScreen databases, totaling around 4.15 million compounds [45]. The most potent BChE inhibitor reported, compound **33**, had an IC_50_ of 10.17 μM against huBChE.

A six-feature 3D pharmacophore model developed by Lu et al. was also used to perform virtual screening of the Vistas-M, ChEMBL, and ChemBridge databases [44,69]. The model itself had been generated using a reference complex (PDB: 5DYW) and consists of a pair of hydrogen bond acceptor points and hydrophobic points, along with one aromatic ring point and one positive ionizable point. Using this model, a total of 18,498 compounds with a pharmacophore fit value greater than three were retained from the first filtration. Subsequent filtration was based on fit to the model, Lipinski parameters, Veber rules, docking, and clustering using the Cluster Ligand module of Discovery Studio. Of the fifteen ligands, two were observed to be selective towards BChE. Follow-up molecular dynamics of the two compounds in complex with BChE showed an energetically favorable Gibbs free energy of binding between the ligands and enzyme, calculated with the MM-PBSA method [69]. Residues of the oxyanion hole and CBS were noted by the authors as important for the binding of one of their inhibitors with BChE, while the other is believed to form a salt bridge with residues of the PAS and form π-π interactions with Trp231 of the acyl pocket.

Researchers at the National Institute of Chemistry in Slovenia developed LiSiCA-2015, an open-source software package that performs LBVS [70]. In their proof-of-concept release, the authors decided to use their tool to find BChEIs. LiSiCA works by taking two compounds as input, converting them to their molecular graphs, computing the vertices and edges of their product graph, and finding the subgraph common to both input graphs that contains the greatest number of vertices. They began by choosing the ZINC “Drugs Now” dataset with 7.4 million compounds, followed by filtering PAINS compounds, poorly soluble compounds, and compounds predicted to aggregate. The resultant compounds then underwent LBVS and were ranked by Tanimoto coefficient. Thirty of the top results were then selected for kinetics assays, of which six were discovered to be highly potent huBChEIs, all with IC_50_ values less than 1 µM. However, a parallel study with AChE was not performed, and as such, the selectivity of these compounds for BChE is unknown.

Lu et al. utilized a pharmacophore model as the base step of their hierarchical filtering methodology [71]. The specific pharmacophore model developed was done so through the Discovery Studio module Receptor-Ligand Pharmacophore Generation, where the input was a reference BChE-ligand complex (PDB: 4TPK). The minimum and maximum number of elements for the model were reported as three and six, respectively. The model was used to screen the Enamine compound library, where over 340,000 potential hits were identified. Also included in the methodological hierarchy were molecular docking and MM-GBSA steps, in descending order. Of the ten selected molecules to undergo in vitro kinetics testing following this additional filtering, five showed IC_50_ values < 100µM, and one was found to be highly selective for BChE. This compound was also found to have no measurable toxicity on PC-12 cells in vitro, and the LC_50_ of this compound is >100 µM.

While tacrine is moderately selective for BChE over AChE, the selectivity index is comparably low. Williams et al. developed a pharmacophore based on the binding modes of modified tacrine, seeking to improve upon the tricyclic compound’s selectivity toward BChE (PDB: 4BDS) [31]. The authors added a chlorophenyl group at the cyclohexyl moiety of tacrine to introduce a static clash with Tyr337 and Phe287 in AChE (PDB: 4EY5), then used their model to filter the NCI/DTP Open Chemical Repository, where 275 compounds were selected. The remaining compounds were then docked against both ChEs and **41** compounds whose docking predicted binding affinity was greater for BChE were purchased for in vitro testing. Of these compounds, six with ≥75% inhibition were chosen for kinetics experimentation, where three compounds, **15**, **22,** and **23,** were identified to be sub-50 nM inhibitors of BChE. Compound **15** was identified by the authors as the most promising candidate on account of its relative ease of synthesis. Compounds **22** and **23** were expected to bear similarity to tacrine in the tricyclic moiety, but with additional aromatic substituents on the third non-aromatic ring. Both **22** and **23** adopt an extended, planar conformation in BChE and AChE complexes, while experiencing clashes with residue Tyr337 in AChE [31].

## 5. Selective BChE Inhibitors from qHTS and ML

### 5.1. Quantitative High-Throughput Screening (qHTS)

The qHTS method is a relatively new strategy of in vitro screening pioneered by Inglese et al. in 2006 [72]. Classical High Throughput Screening (HTS) has been in use since the 1990s as a consequence of advances made during that century in the areas of combinatorial chemistry and commercially available compound libraries [73]. However, HTS is not suited for large assays on account of the frequency of false positives and negatives, often requiring costly follow-up testing. While classic HTS only operates at one concentration at a time, qHTS involves screening a compound library prepared as a titration series in order to establish a concentration-response. In the authors’ original paper, a correlation coefficient greater than 0.98 was found when comparing the AC_50_ between each of the runs, highlighting the improved accuracy and precision of qHTS over HTS performed up to that point.

**Figure 8:**
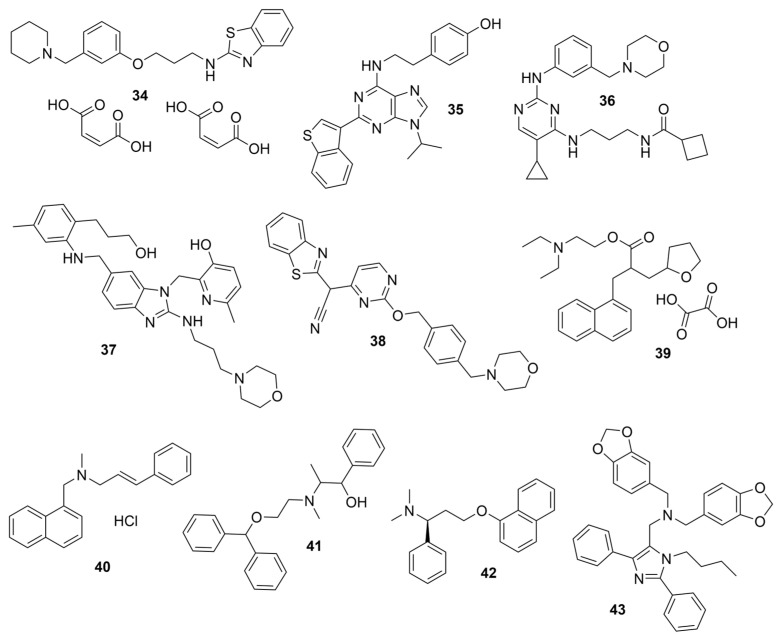
BChE inhibitors and other compounds identified from qHTS.

To our knowledge, not many research groups have performed qHTS in search of BChE-selective anticholinergic agents. Li et al. of the NIH screened 8998 compounds from several annotated libraries against an enzyme-based BChE inhibition assay using qHTS [46]. The compound database includes the Library of Pharmacologically Active Compounds (LOPAC), National Center for Advancing Translational Sciences (NCATS), Pharmacologically Active Chemical Toolbox (NPACT), and NCATS Pharmaceutical Collection (NPC). A total of 125 BChE inhibitors were identified through screening and selected for further study. Researchers hierarchically clustered 125 compounds using Euclidean distance with the complete linkage method based on ToxPrint fingerprints generated within the publicly available ChemoTyper application [46]. The 125 confirmed BChE inhibitors were grouped into several structural clusters with different chemotypes. Of these compounds, a group of purine derivatives, including zolantidine dimaleate (**34**), stemRegenin 1 (**35**), MRT-67307 (**36**), TMC353121 (**37**), and bentamapimod (**38**) were identified as novel BChE inhibitory compounds. In addition, a novel cluster of compounds containing nafronyl oxalate (**39**), naftifine hydrochloride (**40**), difeterol (**41**), dapoxetine (**42**), and NDT 9513727 (**43**) were shown to inhibit BChE. Molecular docking analyses indicated that BChE-selective bentamapimod interacted with His438 and Ser198 residues of the catalytic triad through its benzothiazole moiety but did not bind to key residues in the AChE active site (Figure 8).

Another application of qHTS to the cholinergic selectivity problem came two years later, by Xu et al., who are also affiliated with the NIH [47]. Their tested ligands included the Toxicology in the 21st century (Tox21) 10K compound library, which was docked against AChE, and compound collections including drug and drug-like molecules against BChE. A total of 2195 ligands were shared between the two datasets, where 98 were exclusively active against AChE, 201 were found to be exclusively active against BChE, and 97 inhibitors were listed as nonselective. For either enzyme, the vast majority of compounds tested were found to be inactive. Specifically, 6.8% of compounds tested for AChE activity and 10.5% tested for BChE activity were found to be active. The authors then calculated the max TC values within the active inhibitors and between the active and inactive inhibitors in each ChE data set to evaluate the structural similarity of the datasets. It was found that the average max TC values were markedly greater than those calculated between the active and inactive inhibitors. This result implies that inactive compounds are more structurally distinct from active compounds than active compounds are from themselves.

### 5.2. Machine Learning Models

**Figure 9:**
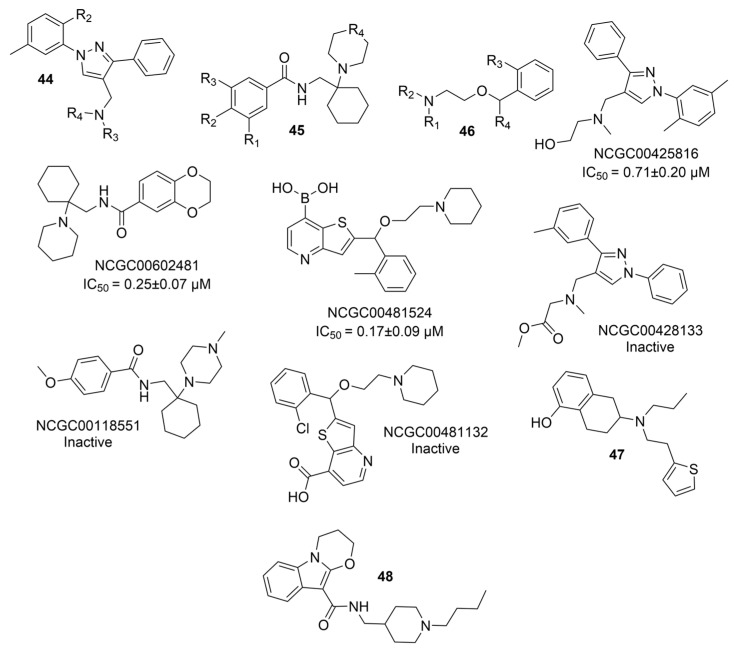
BChE inhibitors and other compounds identified via ML models.

In the past 5 years, machine learning models have increasingly been employed to advance drug discovery, and a few groups have trained models in the hope of improving the effectiveness of in silico prediction of potent and selective ChEIs (Figure 9). Xu et al. extended their qHTS study screening by using machine learning algorithms to build 2D-Quantitative Structure Activity Relationship (2D-QSAR) models and utilized these models to screen an in-house collection of ~360K compounds [47]. The highest performing models achieved AUC-ROC values ranging from 0.83 to 0.87 for the prediction of inhibition activity and selectivity for both ChEs. The BChE selectivity was found to be related to multiple structural features. For instance, the “ring:hetero_[6_6_]_N_S_phenothiazine” descriptor contributes to the BChE selectivity of ethopropazine over AChE by ~1000-fold. Three series of novel compounds were identified as BChE selective: the 1,3-diphenyl-1H-pyrazol-4-yl)methanamine series (**44**), N-((1-(piperidin-1-yl)cyclohexyl)methyl)benzamide series (**45**), and the 2-(1-phenylethoxy)ethan-1-amine series (**46**). Interestingly, inactive inhibitors were also discovered with the same structural moieties in each of the series of compounds listed. With that noted, Xu et al. were able to confirm 68% of their ML-model-predicted BChE inhibitors indeed inhibited BChE through calorimetric and in vitro experimentation.

Li et al. also employed a 2D-QSAR model based on ECFP4 molecular fingerprints with several machine learning algorithms (XGBoost, RF, SVM, KNN), among which the XGBoost model showed the best performance with an AUC of 0.9740 [48]. Following screening with the 2D-QSAR ML model, a hybrid SBVS and LBVS methodology revealed 12 hits from the TopScience core database of 827,897 compounds, three of which were found to be BChEIs in vitro. Among them, rotigotine (**47**) and piboserod (**48**) demonstrated the best BChE inhibitory potency and exhibited favorable safety profiles as well as neuroprotective effects in vitro. Compound **47**, a dopamine agonist approved for Parkinson’s disease treatment, was newly recognized for its anti-AD potential, with further enzyme kinetic analyses revealing that it acts as a mixed-type inhibitor in a non-competitive mode. Both **47** and **48** have a tertiary amine group, which is protonated at physiological pH. Rotigotine, with its tetrahydronphthalene moiety situated deep into the cavity, formed a hydrogen bond with His438 and three π-π interactions with Trp82 and His438 in BChE-**47** complex (PDB: 5DYW). In addition, **47** led to a reduction in RMSF values in the BChE-**47** complex near residues Trp82 and Trp231. Furthermore, the average solvent accessible surface area value of the BChE-**47** complex was slightly lower than that of free BChE, suggesting its binding may cause the protein to be more locally compact.

Ganeshpurkar et al. too had trained a series of ML models in search of BChE inhibitors [74,75]. The authors utilized the BindingDB database containing 7885 known BChEIs. The ligands underwent energy minimization, molecular descriptors of the resulting 3D structure files were generated using RDKit, and highly correlated features were identified and excluded. For their ML algorithm, the authors chose the SVM method for all models trained. Reported ROC AUC for the final ten models lies in a range of ~0.78–0.87. Following a hierarchical workflow including PAINS and property screening, molecular docking-based virtual screening, and 50 ns molecular dynamics simulations, the authors yielded three potential BChEIs. With that noted, these compounds were not tested in vitro to determine the extent of their inhibition. Furthermore, their ML model does not account for AChEIs, and so knowledge of the selectivity of these compounds for either ChE is also unknown.

Another study intended to identify novel ChEIs was performed by Tripathi et al. [76]. Researchers used an in-house library of 210 molecules, and the OCHEM webserver was used to generate descriptors and train the models. A total of 48 models were trained using ASNN, KNN, WEKA-RF, and XGBoost ML algorithms. The best four models following five-fold cross-validation were chosen for a single consensus model. The consensus model had a reported accuracy of ~76% against the test set. The authors then employed SBVS, MM-GBSA, density functional theory, and molecular dynamics to eventually land on a single compound, which was verified as a dual inhibitor in vitro. While this study did not explicitly test BChE selectivity, they reported detailed statistics from their training, which detail the descriptor/ML algorithm combinations that contributed to the highest correlation. Since their models handle AChE and BChE inhibition predictions separately, these statistics may help shed light on effective strategies for future selectivity-oriented model development.

Recently, Ozalp et al. developed contrastive learning and deep learning models for the purposes of identifying BChEIs [77]. Working from the ChEMBL human BChE dataset, researchers used ECFP fingerprints for the training of single and sequential models. The reported AUC scores on the validation set corresponding to the BChE > AChE sequential model for BChE selectivity are 0.76, 0.69, and 0.72 for the CL, DL, and RF models, respectively. Using the sequential models, 5 million compounds underwent VS. Following filtering of the hits using MegaTox and excluding ligands that are not purchasable or easily synthesized, the authors settled on 20 compounds for experimental analysis of binding inhibition. Though none of the tested molecules had a percent relative inhibition greater than rivastigmine for BChE, each of the 20 compounds favored BChE over AChE. Furthermore, seven of the compounds had a relative inhibition of over 30%, resulting in an accuracy across models of 35% when using 30% as a baseline. The DL method had the highest accuracy of the three models they tested, with 43%, though only 7 molecules were tested from this model.

## 6. Discussion

### 6.1. Hits from Traditional Methods

**Figure 10:**
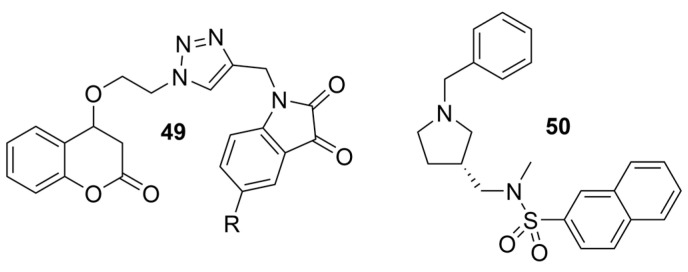
BChE selective inhibitors for the discussion of hits from traditional methods.

Even with a wide range of options for screening large compound libraries, traditional approaches still play an important role in drug discovery today. Nature presents abundant biologically active resources from which we can isolate compounds and identify potential therapeutics [78,79,80,81,82,83,84], while the demand for specific features/scaffolds may require synthetic approaches to obtain, modify, and create, sometimes even to purify in a specific enantiomeric format [85]. One example that exemplifies the use of these methods is a novel Coumarin–Triazole–Isatin hybrid **49** (Figure 10), which has been found to have a BChE selectivity index of 8.78 [49]. In the hBChE-**49** (PDB: 5K5E) complex, the coumarin moiety engaged in a π–π stacking interaction with Trp82. Moreover, Phe329 formed a π--alkyl interaction with the isatin core, while Ser198 established a hydrogen bond with the carbonyl oxygen at position 3 of the ring. The isatin heterocycle engages in interactions with residues of both the CAS and PAS due to the relative flexibility of BChE’s active site [49]. In the *Tc*AChE-**49** (PDB: 5NAP) complex, the coumarin moiety established a π–π stacking interaction with Trp84 from CAS and an H-bond with Tyr130 of the CAS. In addition, Phe330 from the aromatic cluster near the CAS engaged in π–π stacking with the benzene ring of the isatin core. Both the triazole ring and the diketone ring of isatin form a π-donor hydrogen bond with Tyr121.

Traditional methods may also be employed to improve the absorption of a given drug candidate to relevant biological compartments. For example, to overcome modest brain exposure of the BChE-selective racemic compound **31**, an R-enantiomer of analogue **50** (Figure 10) was developed, which has 7-fold higher in vivo brain exposure than **31** [50]. As noted, endogenous BChE is present in a larger concentration than AChE, and fine-tuning compounds to maximize uptake to the brain and avoid non-essential compartments is critical for the transition from BChEI to a legitimate drug candidate. Compound **50** produces no cholinergic adverse effects or motor deficits and has no acute toxic effects in mice reported. Compound (S)-(+)-**50** (PDB: 9I02) forms a somewhat weaker interaction characterized by a water-sulfonyl oxygen distance of 3.9 Å compared to 3.3 Å for compound (R)-(−)-**50** (PDB: 9I03). This has been suggested as the reasoning behind why (R)-(−)-**50** (Ki = 6.6 nM) is a more potent huBChE inhibitor than (S)-(+)-**50** (Ki = 14.3 nM).

### 6.2. Hits from In Silico VS Methods

The development of large databases of compounds and powerful computational resources enables virtual screening to be a valuable method for drug discovery to this day. As we have discussed thus far in this document, a number of VS methods often result in an overabundance of hits, requiring further filtration to obtain a reasonable set of ligands that can be purchased or synthesized. Thus, ideas obtained from traditional methods are useful to narrow down the size of search databases or modify hits from VS to enhance their inhibitory potential. A number of authors have utilized both methodologies in their identification of inhibitors [86,87,88,89,90,91,92,93,94,95,96,97,98,99,100,101]. For example, compounds **15**, **22**, and **23** do not have tacrine moieties in their structures, but they are found to have similar binding interactions when inspecting the tacrine docking poses in BChE and AChE. The addition of two large hydrophobic chlorophenyl groups on the cyclohexyl moiety of tacrine increases binding energy from −8.1 kcal/mol to −9.6 kcal/mol for BChE and introduces several clashes with the aromatic residues in AChE, namely Phe297 of the acyl pocket and Tyr337 of the CBS.

The phenothiazine derivatives possess biological anti-ChE properties according to traditional drug discovery methods. Compound **24** was a hit from a virtual screening of phenothiazine derivatives. This compound has a tertiary amine group in the N-methyl-2-piperidinethyl substituent moiety, which the authors reason will bind at the CBS once protonated at physiological pH. The short linker to a rigid pentacyclic azaphenothiazine ring can be expected to cause problems when binding to AChE. However, BChE selectivity is not significant for this compound, with a selectivity index of ~3.0. Compound **25** was discovered from virtual screening of a library of 382 compounds with a quaternary ammonium moiety in the ZINC database. Compound **25** has an impressive BChE selectivity index (>100), which is attributed to an intermolecular hydrogen bond when binding with huBChE by researchers.

As noted, GNT is a natural alkaloid of plant origin. Galantamine and derivatives are almost exclusively AChE selective due to the quaternary ammonium moiety at biological pH. Alkaloids with solanaceous scaffolds, such as **27,** are predominantly BChE selective. Docking analyses revealed that a hydrogen bond is formed with the catalytic triad, which makes it difficult for the ammonium ion to interact at the CBS. More importantly, the relatively rigid scaffold of **27** could not bind favorably within the hydrophobic acyl pocket in AChE but could so in BChE. Compound **28** is a dual inhibitor against AChE and BChE, which was identified by screening a histamine H_3_-receptor library. Its activity agrees well with traditional pharmacophore reasoning.

The 9-phenylacridinedione (9-PAD) scaffold resembles tacrine and acridine, but the replacement of an amine group by a phenyl group both increases the rigidity and polar surface volume of 9-PAD. Compound **29** becomes BChE selective because it is possible to have both a hydrogen bonding interaction with the catalytic triad and a π-π interaction with the CBS site in the BChE-**31** complex. Compounds **30**, **31**, **32**, and **33** are BChE inhibitors identified by LBVS methods based on ROCS spatial features of the BChE-DPZ complex. Donepezil stretches across the catalytic gorge, which is ideal for AChE, but these compounds are often in more compact, folded conformations than when in complex with BChE. This induced fit situation is only possible in BChE due to the aforementioned differences of the aromatic residues in the acyl pockets of the two ChEs. Compound **26** contains a tertiary amine group, heterocyclic rings, and other features that contribute to inhibitor potency. BChE selectivity for this compound may also come from the availability of a compact folding conformation.

### 6.3. Hits from qHTS and ML Models

The qHTS method offers an impressive alternative to classical HTS for the in vitro screening of large compound libraries. If the libraries are pharmaceuticals, hits may discover previously unknown side reactions or newfound therapeutic applications. Two new classes of compounds to selectively inhibit BChE were identified in [46] (Figure 4): purine derivatives and nafronyl oxalate-containing compounds. Among purine derivatives, only **35** retains a purine ring, while the others (**34**, **36**, **37**, **38**) possess heterocyclic rings with partial similarity to purine. With that noted, all of these ligands have a tertiary amine group. The rigidity of the purine ring in **35**, along with large and adjacent substitutes, may contribute to its BChE selectivity. If the other purine derivatives use the tertiary amine group to bind the CBS site, the rest of the longer substituents attach to this moiety in the opposite direction to those in DPZ. If the purine moiety binds to the CBS site, this tertiary amine group will tend to interact with the PAS site. The nafronyl oxalate **39** and analogues **40**, **41**, **42**, and **43** all have a tertiary amine group, which is far from the center of the molecules, are heavily substituted, or are linked to large substitutes with a short linker.

ML approaches were used to identify structural features for BChE selectivity relative to AChE. Some of these structural features include a piperidine ring, a cyclic CC(=O)C, a steroid moiety, an azo-seven-membered-ring, and an N,S-phenothiazine. However, the same scaffold for **44**, **45**, and **46** produces both active and inactive BChE inhibitors [47], indicating that site-specific binding interactions beyond those identified contribute to the final selectivity. For example, inactive **44** lacks a hydroxyl group to form a hydrogen bond with the triad, while inactive **45** and **46** may have inferior binding poses due to the combination of the hydrogen binding moiety and the tertiary amine group. Similar arguments are applicable for the BChE selectivity of **47** and **48**, though the authors did not elucidate on this aspect.

In [47], however, even though the best model has a reported accuracy of 0.97 AUC on the test set, only one-third of compounds tested in bioassay exhibited inhibitory behavior. This is very similar to results obtained by reference [48], where there is a reported AUC of 0.87 with the test data, yet only 23.3% of model predicted AChE inhibitors turned out to be AChE inhibitors, 68% of model predicted BChE inhibitors were BChE inhibitors, 8.9% of model predicted AChE selective compounds were AChE selective, and 44% of model predicted BChE selective inhibitors were BChE selective. Despite great correlation to the test set, which contains results similar to the training set, neither method has been shown to predict inhibitors with a correlation to experiment over 50%, bar unselective BChE inhibitors in [48] at 68%. As such, these models still have room to improve. Additionally, while many of the other models discussed in this review also have high AOC, no models achieve an accuracy of over 50% when predicted compounds are then tested experimentally for their inhibition. With that noted, work continues to improve ML performance by integrating human intestinal absorption values, the growing list of known BChE inhibitors and their corresponding experimentally found IC_50_ values, and the impact of ML models has already produced promising results with relatively few efforts documented to this point [102].

## 7. Conclusions

Recent developments in the identification of selective BChEIs via traditional and virtual screening approaches are discussed. While both methodologies have yielded a large number of inhibitors over the years, VS methodologies have been the most effective at the identification of novel inhibitors, scaffolds, and lead-type compounds. With that said, a number of these inhibitors are unoptimized, and further optimization of their structure through traditional synthetic-driven methods can greatly improve upon the potency and selectivity of the hits obtained through VS. Thirty-three years on from their initial publication, the three domains identified by Radić et al. remain relevant today [15]. The greatest difference between ChEs is the variance in the residues of their respective acyl pockets, where two phenylalanine residues in AChE are replaced by valine and leucine. Further consideration of the CBS and a key tryptophan in AChE is also relevant to selectivity between ChEs, amongst others. Multitargeted ligands, i.e., those which target both the CAS and PAS, are another promising approach, given they contain a short linker to a bulky substituent, which will drive selectivity away from AChE.

A number of VS options have been employed in the search for BChEIs. Often, combinations of VS methods are used in a workflow to filter the large number of hits that may result. While successes have emerged, the predictive accuracy of these methods could be improved. By this, we mean that when predicted inhibitors are finally tested in vitro, the number of confirmed hits is often less than 40%. The incorporation of models trained via ML algorithms on datasets of in vitro kinetics and inhibition data is a promising direction for the identification of selective BChEIs. One study by researchers at the NIH yielded a 68% success rate for determining BChEIs, though selective BChEIs were obtained with a 44% rate of success [47]. Many ML studies report high correlation when tested against the validation set, though correlation when testing those predicted inhibitors is often lower. This highlights the nontrivial task of training a model via ML algorithms to correctly predict this niche biophysical question, where overfitting, correlation of descriptors used to train the model, and a host of other considerations may affect model accuracy. Nevertheless, screening with ML models in accordance with other VS screening methods in a hierarchical filtering methodology is an ever-improving approach to identifying selective BChEIs.

Another consideration, especially important for the development of highly selective and potent BChEIs, is their potential hepatotoxicity. As stated above, BChE is primarily synthesized in the liver and often makes an excellent biomarker for liver health due to its high concentration in blood. Furthermore, the only FDA-approved small molecule inhibitor that was selective for BChE, tacrine, was discontinued for resulting in liver failure amongst a statistically significant number of patients. There are not many VS options that have been developed to date to screen out potential hepatotoxic inhibitors, specifically, though general toxicology screens do exist. As a supplement to kinetics experimentation, some researchers have begun to test confirmed BChEIs for their potential hepatotoxicity in vitro. While the identification of selective BChEIs is important on its own, ensuring these inhibitors do not negatively impact liver health, in addition to other toxicity considerations, is a critical factor in determining whether the compound identified could feasibly be utilized as an anti-AD therapeutic to be received by humans [103].

## Figures and Tables

**Table 1:** The inhibition constant at 50% (IC_50_) of past and present FDA-approved drugs for the treatment of Alzheimer’s disease and other compounds.

Compounds	Structure	AChE	BChE	Ref.
**benzgalantamine**	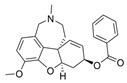			[12]
**donepezil**	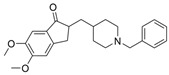	5.7 nM ^a^	7.1 µM ^b^	[17]
**galantamine**	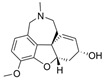	0.36 µM ^a^	19 µM ^b^	[17]
**rivastigmine**	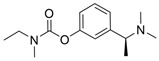	48 µM ^a^	54 µM ^b^	[17]
**tacrine**	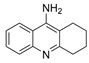	190 nM ^a^	47 nM ^b^	[17]
**1**	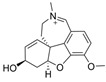	0.14 µM ^c^		[20]
**2**	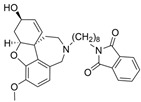	0.28 µM ^c^		[20]
**3**	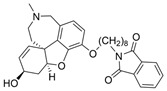	2.50 µM ^c^		[20]
**4**	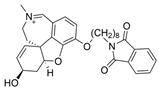	0.07 µM ^c^		[20]
**5**	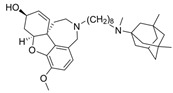	0.52 nM ^d^		[21]
**6**	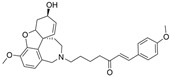	n.a. ^e^	n.a. ^e^	[22]
**7**	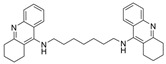	0.4 nM ^d^		[23]
**8**	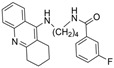	41.37 ± 5.94 nM	1.39 ± 0.17 nM	[24]
**9**	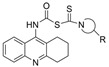	68.39 µM ^a^	0.014 µM ^a^	[25]
**11**	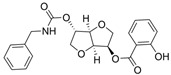	>100 µM ^a^	0.15 nM ^a^	[26]
**12**	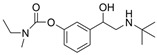	>100 µM ^c^	0.37 ± 0.02 µM ^f^	[27]
**10**	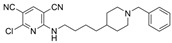	13 ± 2 nM ^a^	8.1 µM ^a^	[28]
**13**	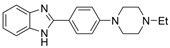	34.83 ± 1.17 µM	5.18 ± 1.22 µM	[29]
**14**	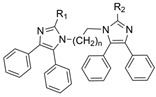	Inactive ^c^	0.03–33.25 µM ^f^	[30]
**15**	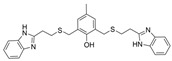	>5000 nM	32 nM	[31]
**22**	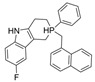	2111 nM	10 nM	[31]
**23**	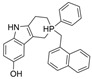	2604 nM	40 nM	[31]
**16**	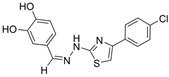	21.3 ± 0.05 µM	1.59 ± 0.01 µM	[32]
**17**	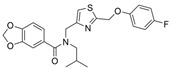	^g^	0.13 µM	[33]
**18**	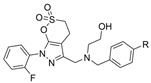	>20 µM	7.7 nM	[34]
**19**	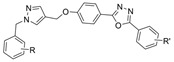	>100 µM ^c^	11.01 µM ^f^	[35]
**20**	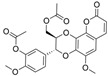		40.1 ± 0.11 µM ^b^	[36]
**21**	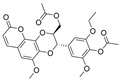		55.4 ± 0.17 µM ^b^	[36]
**24**	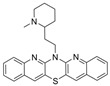	^h^	11.78 ± 1.31 nM	[37]
**25**	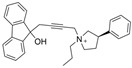	92–762 µM	0.75 ± 0.18 µM ^c^	[38]
**26**	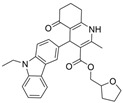	Inactive ^a,i^	0.443 ± 0.038 µM ^b^	[39]
**27**	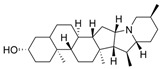	>10 µM ^a^	16.8 nM ^b^	[40]
**28**	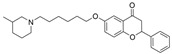	0.36 µM ^a^	0.76 µM ^f^	[41]
**29**	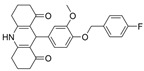	125 µM ^a^	98 nM ^b^	[42]
**30**	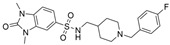	2.05 µM	0.031 ± 0.006 µM	[43]
**31**	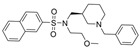	>10 µM	0.049 µM	[44]
**32**	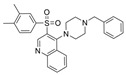		1.3 µM	[44]
**33**	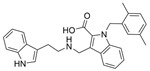		10.17 µM	[45]
**38**	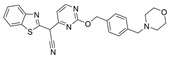	Inactive	0.33 ± 0.02 µM	[46]
**43**	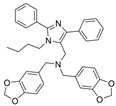	Inactive	0.21 ± 0.01 µM	[46]
**44a**	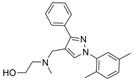		0.71 ± 0.20 µM	[47]
**44b**	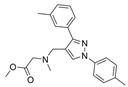		Inactive	[47]
**45a**	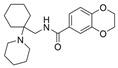		0.25 ± 0.07 µM	[47]
**45b**	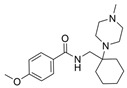		Inactive	[47]
**46a**	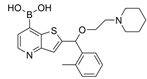		0.17 ± 0.09 µM	[47]
**46b**	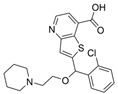		Inactive	[47]
**47**	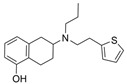		15.33 ± 3.83 µM ^f^	[48]
**48**	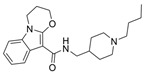		12.76 ± 4.22 µM ^f^	[48]
**49**	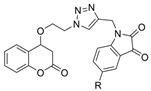	15.28 ± 1.22 µM	1.74 ± 0.29 µM	[49]
**50**	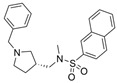	8.24 ± 2.31 µM ^a^	0.013 ± 0.002 µM ^b^	[50]

^a^ human AChE; ^b^ human BChE; ^c^ *Electrophorous electric* AChE; ^d^ murine AChE; ^e^ MD simulation and synthesis; ^f^ equine BChE; ^g^ percent inhibition (%) = −0.92 at 10 µM; ^h^ At 10 mM concentration, **24** inhibits enzyme activity 99.76 ± 0.03% for BChE and 34.77 ± 0.31% for AChE; ^i^ Inactive at 10 µM.
